# Colorectal cancer screening: will non-invasive procedures triumph?

**DOI:** 10.1186/gm562

**Published:** 2014-06-10

**Authors:** Timothy Church

**Affiliations:** 1 Division of Environmental Health Sciences, School of Public Health, 420 Delaware St SE, MMC 807, Rm1260, 55455 Minneapolis, Minnesota, USA

## Abstract

In the US, colorectal cancer is the fourth most common cancer and the second most deadly. Screening is recommended, not only to reduce mortality, but to prevent cancer by detecting precancerous polyps. Many screening methods are available now, and newer methods based on molecular markers show promise for the future.

## Why screen for colorectal cancer?

In the US, colorectal cancer is the fourth most common cancer, after breast, prostate, and lung cancers, and the second most deadly cancer after lung cancer; it will kill an estimated 50,310 Americans in 2014 [1]. Worldwide there are 1.4 million cases per year, making it the world's third most common cancer. But there is good news. In the US among people 50 years old and over, colorectal cancer incidence has been declining by 1.8% per year and mortality by 2.8% per year [1]. This is due to many factors, including improved treatments and adoption of healthier diets and other behaviors in this age group. But a large, and perhaps the largest, factor is widespread adoption of colorectal cancer screening among that age group. Almost two-thirds of that age group report using some form of colorectal cancer screening [1].

Colorectal cancer screening was first evaluated by a study in Minnesota that started in 1975 and was published in the *New England Journal of Medicine* in 1993 [2]. The investigators reported that after 13 years of follow-up, the group offered annual fecal occult blood testing (FOBT) had a 33% lower rate of death from colorectal cancer than the group not being offered any screening (the controls). A third group offered biennial (every 2 years) screening did not show any reduction at that time. However, 6 years later, after screening had stopped, the annual group still had a 33% lower mortality but the biennial group now showed a 21% lower mortality than the controls. Two other randomized trials of FOBT screening, one in the UK [3] and one in Denmark [4], studied biennial screening only and in 1996 confirmed reduced mortality levels in *The Lancet*.

The original Minnesota trial reported in a later *New England Journal of Medicine* paper [5] that annual and biennial screening with FOBT led to 20% and 17% lower incidence (as well as lower mortality) of colorectal cancer, respectively, after 18 years of follow-up. More recently, three other randomized trials of colorectal cancer screening, using an endoscopic method called flexible sigmoidoscopy instead of FOBT, have also reported reductions in both colorectal cancer incidence and mortality using a single screen or two screens 3 to 5 years apart [6-8]. From these trials it is clear that colorectal cancer screening has the capacity to lower both incidence and mortality.

## What are the current recommendations and are they effective?

In the US, several groups, including the US Preventive Services Task Force, the American Cancer Society, and the American College of Gastroenterology, have published guidelines for colorectal cancer screening [9]. They currently all recommend screening beginning at age 50 by one of three methods: colonoscopy every 10 years, flexible sigmoidoscopy every 5 years, or FOBT every year.

FOBT is a test of stool samples for the presence of blood. Some are simple chemical tests (guaiac FOBT) and some are fecal immunochemical tests (FIT). Either form of FOBT is acceptable as long as it is sensitive enough to the presence of blood. The presence of blood is considered a positive test and indicates that the patient may have cancer or advanced pre-cancerous adenomas and requires follow-up with colonoscopy. Colonoscopy is an endoscopic method used to visualize the whole colorectum. Flexible sigmoidoscopy examines only the lower part of the colon and rectum, and if cancer or adenomas are found, follow-up with colonoscopy is also required to complete the examination. Although randomized trials have not been done to show colonoscopy's benefits, the efficacy of both FOBT and flexible sigmoidoscopy depend on colonoscopy to follow up findings on those tests.

Other tests that are available but not universally recommended are double-contrast barium-enema X-rays; 'virtual colonoscopy', a method of using computed tomography to visualize the colon using contrast media; and stool DNA tests that look for additional molecular biomarkers in the stool as well as occult blood. As more is learned about these methods, they may be more widely adopted.

The effectiveness of colorectal cancer screening so far is demonstrated not only by the decline in mortality that exceeds what is expected from improved treatment, but also by the steady reductions in incidence that have been observed in the US since 2006. However, about a third of the people who can benefit from early detection of colorectal cancer are not getting screened [10], and some of them are suffering and dying needlessly.

## Recently, several large-scale studies have examined colorectal cancer screening. What did they find?

Three recent studies, all in the *New England Journal of Medicine *[11-13], that received considerable attention have shifted our understanding of screening. The first was an analysis of the long-term effects of FOBT screening on colorectal cancer mortality in the original Minnesota trial [13]. That report found that the effects observed at 18 years have continued through 30 years of follow-up, even though the study-directed screening ended in 1993. This somewhat unexpected finding demonstrates that the impact of screening may last much longer than we had assumed, and this raises its value in preventing death and disease. In addition, the longer follow-up provided more power to look at the effects of screening within subgroups by age and sex, and found that there may be more benefit of earlier screening for men, whereas the benefit for women may be from later screening at an age older than 50.

The second study used a cohort of individuals some of whom had undergone sigmoidoscopy or colonoscopy during the 20-year follow-up period to evaluate the impact of that procedure on incidence and mortality from colorectal cancer [11]. Although such an approach does not eliminate the potential for biases from self-selection or lead-time, the investigators found evidence of a reduction in both incidence and mortality among those who underwent endoscopy compared with those who did not. In addition, there was evidence that cancers diagnosed within 5 years of endoscopy were more likely to be of the CpG island methylator phenotype than those diagnosed more than 5 years later [11]. This has implications not only for adjusting surveillance periods based on evaluation of pre-cancerous lesions, but also for the development of molecular markers for early detection.

The third, most recent, study evaluated a test of DNA markers in stool [12]. Stools were collected and evaluated from nearly 10,000 individuals. A panel of DNA markers plus an FIT was compared with a stand-alone FIT for sensitivity to colorectal cancers and for advanced adenomas, and the DNA panel was found to be significantly more sensitive, though less specific (that is, it was more likely to identify a lesion, but was also more likely to misidentify a person without lesions as being positive for a lesion). A Food and Drug Administration panel has recently recommended approval of the test for early detection.

## What needs to be done to improve screening?

How to increase the uptake of colorectal cancer screening in the asymptomatic population for which it is intended has been the subject of much research. Unlike other cancers for which early detection is recommended, colorectal cancer offers a number of choices for screening methods. This is both a blessing and a curse: a blessing because it allows flexibility in addressing the preferences and concerns of an individual considering screening, but a curse because it makes the public health message about screening less tidy and potentially more confusing [14,15]. A range of strategies have been studied, from directly mailing FOBT kits to screen eligible people to assigning 'navigators' for people who may be challenged in negotiating the options and restrictions of their own healthcare system. At this point, there is still much to be learned, as the proportion of the population using screening seems to have reached a plateau.

## Non-invasive, genomic methods are often mentioned as the way to increase screening. What are they and will they improve uptake?

Increasing the performance of non-endoscopic screening methods as well as making them more acceptable to the individual are the primary benefits of genomic, epigenomic, and other molecular early detection markers. The stool DNA test in the recent *New England Journal of Medicine* paper detailed above [12] uses a panel of nine different markers (two hypermethylation markers and seven *KRAS* point mutations in addition to an FIT. Several groups are working on molecular markers that can be found in blood, including proteins such as carcinoembryonic antigen, mutated genes such as *KRAS *[16]; hypermethylated genes such as *SEPT9* (encoding septin 9) [17]; and microRNA [18].

## What does the future hold?

Although to date none of these blood markers has been proven adequate for screening, there is a good chance that one or a panel of such markers will eventually be discovered that is sensitive and specific enough for screening, much like what was done in the stool DNA test. Many believe that a blood test would help boost uptake of screening by providing an alternative to those who want neither endoscopy nor a stool test. The robust and rapid uptake of prostate-specific antigen (PSA) testing for prostate cancer despite the lack of clear evidence of efficacy can, in part, be attributed to the fact that it is a blood test and can be administered as a routine part of a standard physical in which blood is taken for cholesterol, glucose, and other substances. A similar test for colorectal cancer with the added benefit of clear evidence of efficacy should drive uptake of screening closer to the ideal 100% of the 50-and-over asymptomatic population. Given the rate at which molecular methods are becoming more and more precise and sensitive, I am optimistic that this goal is not far off.
