# Erratum to: Colorectal cancer screening: will non-invasive procedures triumph?

**DOI:** 10.1186/s13073-015-0206-7

**Published:** 2015-09-07

**Authors:** Timothy Church

**Affiliations:** Division of Environmental Health Sciences, School of Public Health, 420 Delaware St SE, MMC 807, Rm1260, 55455 Minneapolis, Minnesota USA

## Erratum

Unfortunately, the original version of this article [1] published with an incorrect citation. The article number has now been updated to **6**:125, which is the correct citation for the article. The Publisher apologizes for any inconvenience caused.
